# Cortical Modulation of the Transient Visual Response at Thalamic Level: A TMS Study

**DOI:** 10.1371/journal.pone.0017041

**Published:** 2011-02-10

**Authors:** Nelson Espinosa, Jorge Mariño, Carmen de Labra, Javier Cudeiro

**Affiliations:** Neuroscience and Motor Control Group (NEUROcom) and Biomedical Institute of A Coruña (INIBIC), University of A Coruña, A Coruña, Spain; Rutgers University, United States of America

## Abstract

The transient visual response of feline dorsal lateral geniculate nucleus (dLGN) cells was studied under control conditions and during the application of repetitive transcranial magnetic stimulation at 1 Hz (rTMS@1Hz) on the primary visual cortex (V1). The results show that rTMS@1Hz modulates the firing mode of Y cells, inducing an increase in burst spikes and a decrease in tonic firing. On the other hand, rTMS@1Hz modifies the spatiotemporal characteristics of receptive fields of X cells, inducing a delay and a decrease of the peak response, and a change of the surround/center amplitude ratio of RF profiles. These results indicate that V1 controls the activity of the visual thalamus in a different way in the X and Y pathways, and that this feedback control is consistent with functional roles associated with each cell type.

## Introduction

Early research on the role of visual cortical feedback has suggested both a facilitating [1], [2] and an inhibitory effect on thalamic neurons [3], [4]. Other studies have attributed different functions to cortical input, such as the ability to synchronize the neurons of the thalamus, to interfere in the spatial processing of the image or to enhance the receptive field (RF) center-surround antagonism of thalamic cells [4]–[6].

To date a variety of methods have been used to reveal the role of cortical feedback to the dLGN including pharmacological manipulation, cooling and aspiration of cortical tissue. Recently, repetitive transcranial magnetic stimulation (rTMS) has emerged as an alternative method. It is a noninvasive technique, its effects are fast and its application, depending on the stimulation parameters, allows excitation or depression of cortical activity [7]–[9]. Moreover, it has been shown that rTMS is a valuable tool to study the role of the corticofugal feedback since it is able to transiently reduce the activity of visual cortical neurons [10], [11]. For instance, rTMS at the frequency of 1 Hz applied to the visual cortex of the cat, produced a significant reduction in visually driven responses recorded simultaneously in layer 6 of V1 (the source of the feedback) and in the dLGN [10] (see also methods section).

It has been shown that visual cortex influences receptive field organization in the thalamus [12]. An immediate question is whether this feedback is able to modulate the spatio-temporal properties of dLGN receptive fields as could be expected from what has been said so far and from previous data showing that cortico-geniculate feedback seems to improve the temporal accuracy of signal transmission [13], [14]. On the other hand, it is already known that corticofugal feedback can influence the firing pattern in dLGN cells [12], [15]–[17] and that thalamic cells in general, including those in the dLGN, exhibit two distinct response patterns, burst and tonic firing. However it is not known whether this influence operates in an unspecific manner at the level of the dLGN or, conversely, it acts differentially depending upon the cell type, which would represent the presence of, at least, two (X and Y) physiologically different descending lines of control.

This paper aims to analyze the contribution of feedback from V1 to the spatio-temporal characteristics of RFs and response mode of dLGN cells during visual stimulation. rTMS was applied on the occipital cortex of anesthetized cats to temporarily block the cortical activity [10]. We have chosen to use a specific type of visual stimulus (pseudo-random sequence of bright and dark bars flashed throughout the receptive field) appropriate to map the spatiotemporal RFs of dLGN cells [18] and valuable to evoke robust transient responses of spike bursts.

## Results

A population of 51 dLGN neurons, 18 X, 28 Y and 5 unclassified cells were recorded. All cells RFs were located within 12° of the *area centralis*.

### Burst analysis

We calculated the number of spikes per burst and the percentage of spikes in bursts, and found no significant changes after applying rTMS@1Hz when all the dLGN cells were grouped together (X, Y and unclassified), or by dividing the population by polarity (ON - OFF) or laterality (ipsi-and contralateral). However, significant differences between the X and Y subpopulations were evident, with rTMS affecting Y cells but not X cells.

### Burst analysis for X cells

**Figure 1:**
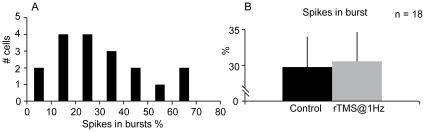
Percentage of spikes in burst mode for X cells. ***A*** *,* Distribution of the percentage of spikes in burst for the response of X dLGN cells. ***B***, In these cells, the percentage of spikes in burst remained unaltered with rTMS@1Hz.

Figure 1A shows the distribution of the percentage of spikes in burst during control condition. Statistical analysis revealed that rTMS@1Hz did not alter either the burst or tonic response, as it is shown in Figure 1B for spikes in burst.

### Burst analysis for Y cells

**Figure 2:**
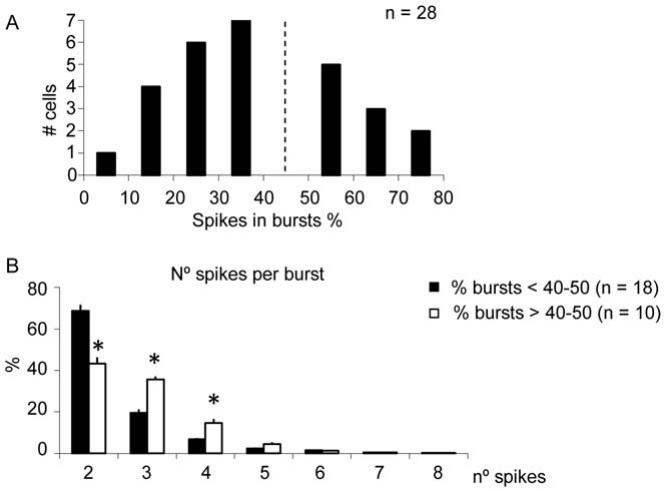
Distribution of the percentage of spikes in burst for the response of Y dLGN cells. ***A***, Cluster analysis splits the population in two groups around 40–50% (dashed line). ***B***, Distribution of the spikes per burst for Y dLGN cells. Neurons whose response had a low content of burst spikes (<40–50%, dark bars) mainly emitted bursts with two spikes. In contrast, cells with a high content of burst spikes (>40–50%, white bars) produced bursts with relatively more spikes. Vertical error bars represent the standard error of the mean.

Burst properties of Y cells were not affected by the application of rTMS. However, differences were found depending on their visual response prior to rTMS application. Figure 2A shows the distribution of the percentage of spikes in bursts for Y cells during control conditions. A visual analysis of Figure 2A shows that there are two populations of neurons that generate bursts with different rate. To corroborate this subdivision of the sample, a two-phase clustering was performed, yielding a cut-off value around 40–50%, which divides the sample into neurons with low and high percentages of bursts in the response shown in Figure 2A (dashed line). In addition, an intra-burst analysis revealed that the subgroup with a high rate in burst response tended to generate bursts with a greater number of spikes than cells with a lower rate (Figure 2B, p<0.05, two samples t-test). This result suggests that these groups could constitute two different populations of neurons, henceforth each group was analyzed separately.

**Figure 3:**
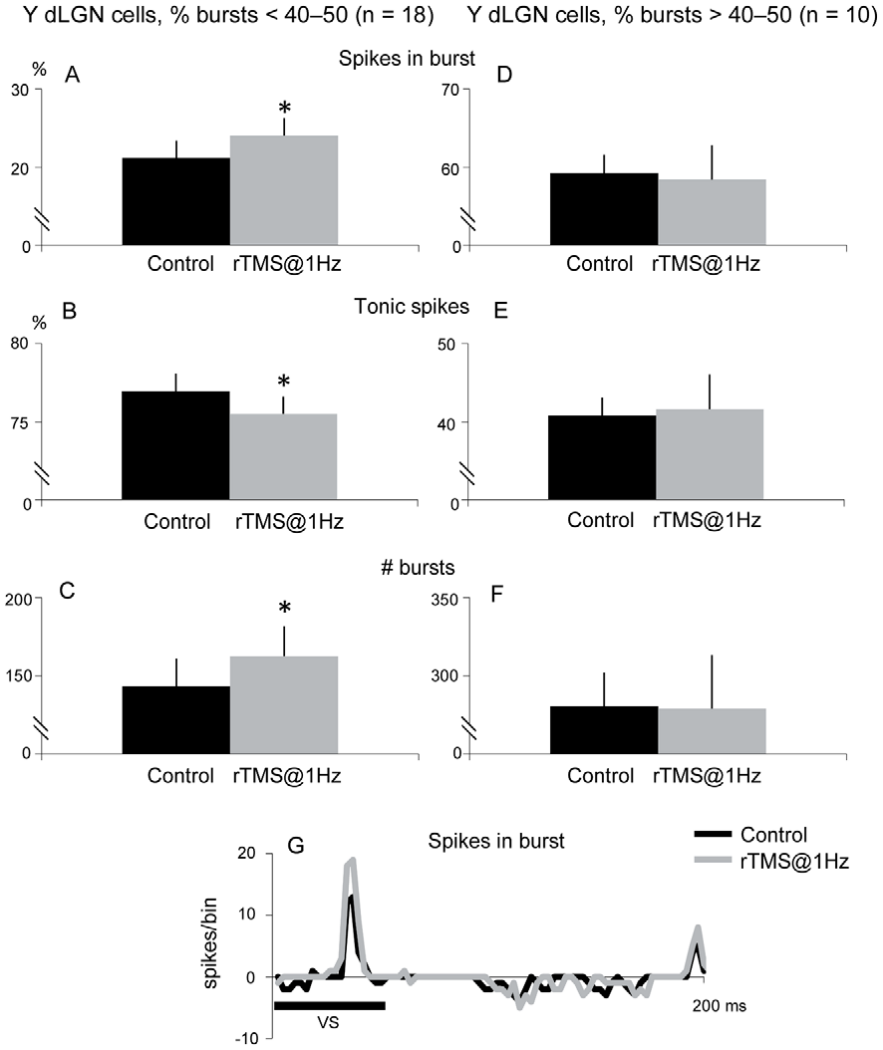
Analysis of Y dLGN cells whose percentage of response in burst was less than 40–50%. rTMS@1Hz increased the percentage of spikes in burst *(* ***A*** *)* and consequently decreased tonic spikes *(* ***B*** *).* Changes in response mode were reflected, as well, in the number of bursts *(* ***C*** *).* ***D*** **–** ***F***, rTMS@1Hz did not alter the response mode of Y dLGN cells whose percentage of response in burst was more than 40–50%. ***G*** *,* An example of a Y dLGN cell response whose percentage of spikes in burst during control condition (black line) was 29%. rTMS@1Hz increased spikes in burst to 38% (gray line, bin = 2 ms). Vertical error bars represent the standard error of the mean.

Neurons with a low percentage of bursts, i.e. the proportion of spikes in bursts was below 40–50% (n = 18), showed a significant increase in the percentage of spikes in bursts when rTMS@1Hz was applied, as it is shown in Figure 3A (21.1±2.2% to 24.0±2.2%, P<0.05, paired t-test) and therefore a decrease in the percentage of tonic spikes (78.9±2.2% to 76.0±2.2%, P<0.05, paired t-test, Figure 3B). Considering each burst as an event, rTMS@1Hz application induced an 11.0% increase in the number of bursts (from 143.2±17.7 to 162.5±19.0, P<0.05, paired t-test, Figure 3C).

Figure 3G shows an example of an ON Y cell with a low rate of spikes in bursts during control (29%, black line). Application of rTMS@1Hz increased the percentage of spikes in bursts to 38% (gray line).

Neurons with a high percentage of bursts, i.e. the proportion of spikes in bursts were more than 40–50% of the total response (n = 10), did not show significant changes with rTMS@1Hz application (see Figures 3D–F).

### Intervalogram analysis

Only spikes separated in the range from 0 to 20 ms were considered for the analysis because almost all interspike intervals during the transient phase of dLGN cells response fell into this range (see Material and Methods). According to the previous results, intervalogram analyses were developed separately for X and Y subpopulations.

### Intervalogram analysis for X cells

**Figure 4:**
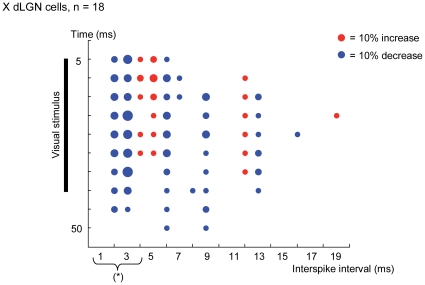
Scatter plot obtained subtracting average intervalogram of rTMS from control condition for X dLGN cells. Blue and red dots represent decrease and increase of interspike intervals (X axis) along the time (Y axis) respectively. Dot size depicts the magnitude of change and black bar shows the duration of visual stimulus. Asterisk indicates the interspike interval range for spikes in burst.

Average intervalograms for X neurons population were calculated for both, control condition and when rTMS@1Hz was applied. Figure 4 shows the scatter plot of the difference between these two intervalograms. Dots indicate an increase (red dots) or decrease (blue dots) in the number of a determined interspike interval and dot size represents the degree of change. During the presentation of visual stimuli (vertical black bar on left) the main changes induced by rTMS are associated with a decrease in the number of spikes separated from 1 to 3 ms. Elsewhere in the intervalogram there are more subtle changes generally related to a decrease of interspike intervals.

### Intervalogram analysis for Y cells

**Figure 5:**
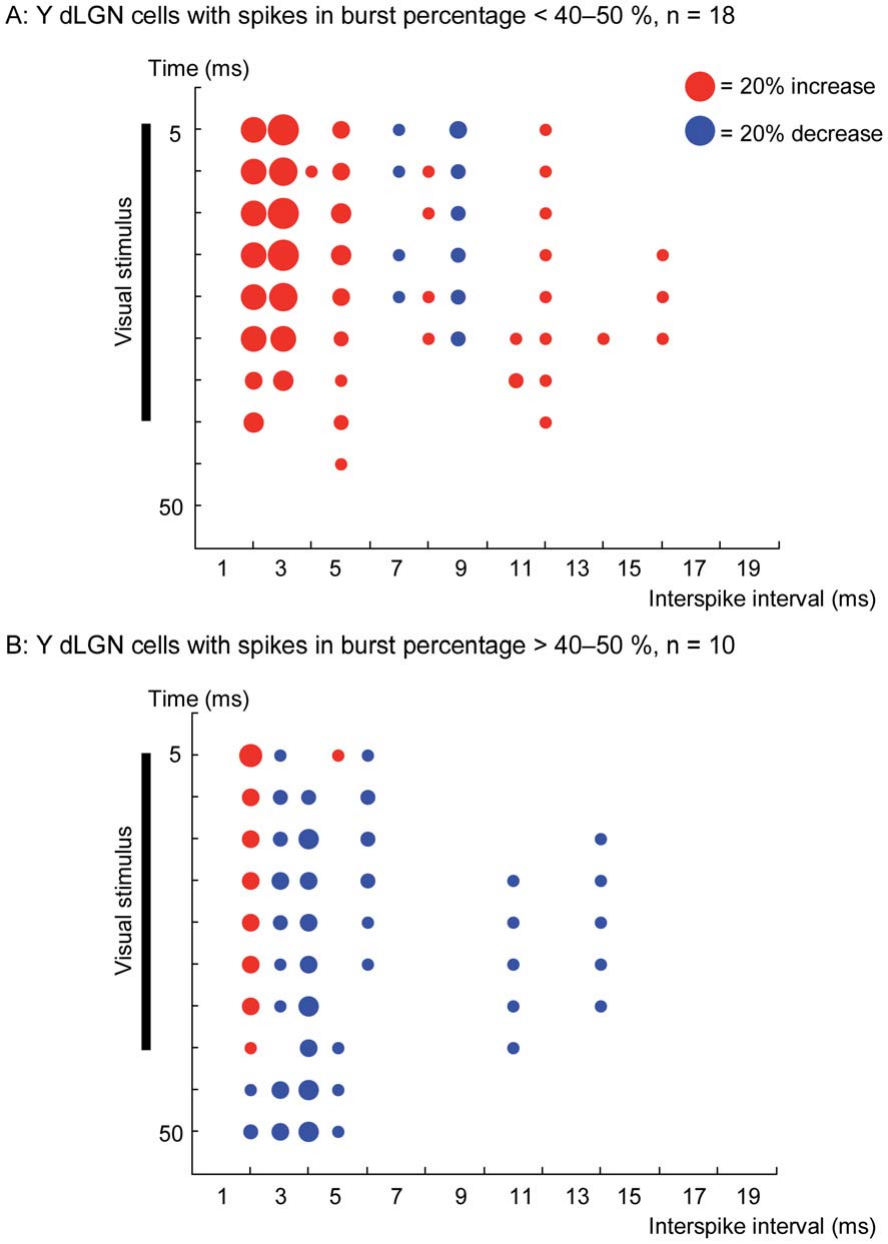
Scatter plot for Y cells. Scatter plot of changes on intervalograms induced by rTMS@1Hz for Y dLGN cells with low *(* ***A*** *)* and high *(* ***B*** *)* content of bursts.

The sample of Y cells was subdivided depending on their rate of bursts response considering our previous bursts analysis. Y neurons with a low rate of spikes in bursts (smaller than 40–50%) increased the amount of interspike intervals within a range of 1 to 5 ms during rTMS application (Figure 5A). In contrast, neurons with a high percentage of bursts seem to be less affected by rTMS@1Hz with a scatter plot that reflects changes of slighter magnitude (Figure 5B).

### Spatiotemporal response analysis

We then studied the spatial and temporal profile of the dLGN cells visual response using the reverse correlation technique. No significant changes were observed when considering the whole population (X, Y and unclassified). The analysis was then performed separately on X and Y cells as follows.

### Spatiotemporal response analysis for X cells

**Figure 6:**
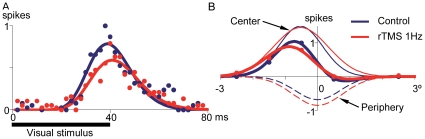
Example of a dLGN X-on cell whose STRF profile was modified by rTMS@1Hz. Circles represent real values normalized to control and curves their mathematical fits. ***A***, Transient response elicited by a white bar presented at the RF center shows a decrease in peak value (from 80 to 60%) and an increase in latency (from 39 to 41 ms) when rTMS was applied. ***B***, Construction of RF profile by fitting a difference of Gaussians (DoG) function to real values. rTMS@1Hz modified the rate between Gaussian amplitudes by a decrease of the center (upper thin curve) and an increase of the periphery (lower thin curves).

**Figure 7:**
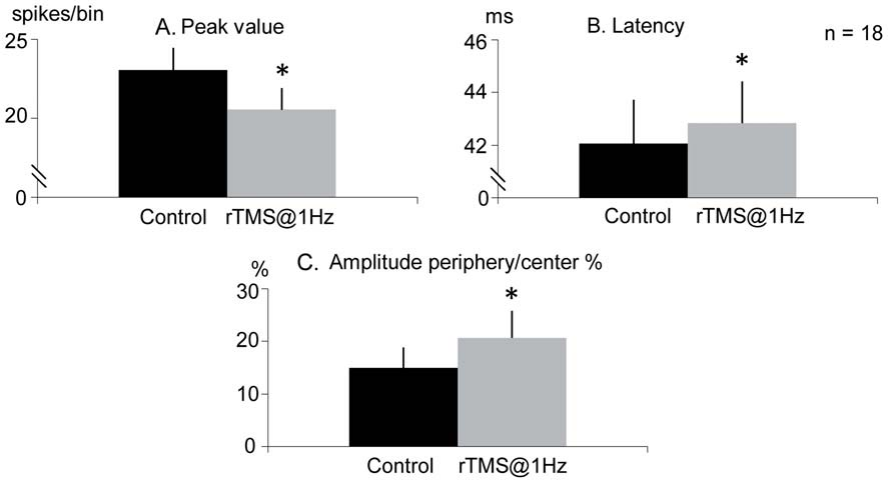
Effect of rTMS on X cells. rTMS@1Hz decreased *(* ***A*** *)* and delayed *(* ***B*** *)* the peak transient response and induced an increase in the rate between periphery and center amplitudes *(* ***C*** *)* in dLGN X-cells (bin = 2 ms). Vertical error bars represent the standard error of the mean.

The application of rTMS@1Hz induced changes in the spatiotemporal response of X cells. Figure 6A is an example of an ON X cell showing a decrease in peak response and an increase in peak latency after applying rTMS@1Hz. The cell also showed a change in the surround/center amplitude ratio (Figure 6B). Figure 7 shows the mean changes induced by rTMS@1Hz in cell responses for the population of X neurons. There is a decrease in the maximum response of 10.9% (from 23.0±1.4 to 20.5±1.4, P<0.05, paired t-test, Figure 7A), a slight increase in latency (42.0±1.6 to 43.0±1.6, P<0.05, paired t-test, Figure 7B) and an increase of 5.8% in the surround/center Gaussian amplitude ratio (from 14.9±4.0% to 20.7±5.0%, P<0.05, paired t-test, Figure 7C).

### Spatiotemporal response analysis for Y cells

The application of rTMS@1Hz did not induce significant changes in the response of Y neurons, either taking all the population of neurons or subdividing the sample according to the percentage of bursts (not shown). It is worthy of notice the fact that, in contrast to the X cell population, a group of Y neurons (n = 11) did show a decrease in latency. The remaining neurons presented an increase (n = 9) or no change in this parameter (n = 8). None of these groups was identified as neurons with a high or low percentage of bursts.

## Discussion

We have applied rTMS@1Hz to explore the influence of the corticofugal pathway in the transient phase of dLGN cells visual response. We have chosen this low-frequency stimulation protocol because it has been shown to be a fast and reliable method to transiently depress the excitability of the cerebral cortex [10], [19] (see example in the method section). Moreover, we have previously used this protocol to demonstrate that corticogeniculate downflow selectively affects the sustained component of the visual response of dLGN cells with no effect on bursts discharge [10]. In the present work we show that the visual cortex exerts a differential control on burst frequency and spatiotemporal RF organization depending on the cell type. This apparent contradiction may be explained taking into account some differences in the visual stimulation protocol. In previous works, to study the whole visual response of ON dLGN cells, we initially presented a black background and subsequently a white dot centered on the RF, and vice versa for OFF dLGN cells [10]. This proved to be an optimal way to analyze the effect of rTMS in the sustained phase of the response, but it had some disadvantages when considering the transient phase. For a relay dLGN neuron, prolonged exposure to a stimulus with a non-preferred contrast (black for an ON cell) produces a "rebound" response at the end of the stimulus mainly constituted by spikes in bursts [20]–[22]. These bursts may be driven by the inhibition produced by dLGN interneurons whose receptive field position coincides with the relay neuron, but with an opposite polarity (ON or OFF interneurons) [22]. Thus, an initial black background presented to an ON dLGN neuron preconditions their Vm to a more hyperpolarized level, by disynaptic inhibition via OFF interneurons, which in turn favors the generation of bursts once the visual stimulus appears. In the present study special care was taken to maintain a stable state in the inhibition that directly depends on retinal input [22]. To do this, a gray background (mean luminance of 45 cd/m^2^) for black and white bars series was used to keep the same background activity for interneurons and relay cells.

To analyze spikes in bursts, we applied the criteria based on intracellular recordings in cat dLGN neurons [23]. These are the most widely accepted criteria for burst analysis of extracellular recordings [12], [24]–[26]. Additionally, we tested a more restrictive selection to prevent the inclusion of high frequency tonic spikes, considering each burst as a train comprised at least of three spikes [10], [17], [27]. Finally, we applied both criteria but considering 6 ms as the maximum intraburst interspike interval [28]. All criteria gave similar results which reinforces our conclusions.

### Effects of rTMS on transient phase response

Previous results [29] showed that cortical cooling induces a decrease in the peak response of the transient phase of on X cells (a decrease of 18%) whilst the peak value of Y cells remained unchanged. Our study adds new information, showing that the cortex selectively influences the spatiotemporal response of X neurons producing, together with a decrease in peak response, a slight increase in peak latency of around 1 ms; both phenomena are probably associated with an imbalance in the center/periphery amplitude ratio. Moreover, burst analysis showed that Y cells, but not X cells, increase their burst rate and reduce tonic spikes when the cortex is inactivated. This behavior was observed in those Y cells characterized by having a burst rate at control conditions of less than 40–50%. This splits our Y cell sample into two groups that also differ in their spikes per burst distribution. Since the number of spikes per burst increases at higher levels of hyperpolarization [30], all these data indicate that cortical blockade influences primarily neurons with some prior level of depolarization. Thus, when cortical input is reduced, dLGN neuron's Vm becomes more negative, increasing the number of spikes per burst. Hence, it is reasonable to hypothesize that Y cells with a high response rate in bursts reflect a lower pre-rTMS cortical activity, a condition in which the TMS seems to generate less effect on the cortex [11]. As far as we know, this clustering of Y cells has not been reported previously, although a study focused on dLGN cells burstiness suggests certain subdivision of Y cells depending on the "burst fraction" of their response [31].

Differences of cortical effects between X and Y cells could be due to some differences of connectivity patterns in both pathways. Besides the fact that both groups of neurons receive inputs from different types of retinal neurons, there is evidence that the X pathway could be exposed to higher local inhibitory control than the Y pathway [32], [33] and that each pathway could have independent interneurons contacting only with the associated relay neurons [34], [35]. Moreover, in visual cortex both pathways remain partially segregated in V1; reviewed in [36], but see [37]. These results suggest the existence of a difference in corticothalamic connectivity, as it has been recently shown by others in monkey [38].

Regarding corticothalamic synapses, neuroanatomical studies have revealed that axons coming from area 17 make contact with a larger number of interneurons (17%) than those arising from area 18 (7%) [39]. This suggests that area 17 would be able to exercise a more powerful control on dLGN neurons than area 18 [29]. Additionally, it has been reported that axon terminals coming from area 17 make contacts preferably with X neurons and, conversely, the fibers from area 18 are more related to Y neurons [39]–[41]. Taken together, these data would imply a greater inhibitory impact from area 17 axons on X cells. Then, it is possible that the net effect on the Vm of X relay neurons depends on the excitation-inhibition balance, being more robust to changes in cortical activity. Although it would be necessary to make an intracellular study, the present results suggest that the cortical blockade indices a stronger effect over the Vm of Y cells than that of X cells.

Finally, it is open to question how X cells, whose burst responses seems to be more stable to changes in cortical activity, decrease and delay their peak response. The answer seems to be related with the type of visual stimulus used. A bar presented in the RF center during 40 ms transiently stimulates both the center and the periphery of the RF. The fact that the action of the periphery is more pronounced in dLGN than in the retina [42], [43] suggests a direct involvement of intrinsic inhibitory processes in the visual response of dLGN neurons. In analyzing the intervalograms during the application of rTMS@1Hz, it appears that X neurons, unlike Y neurons, generated spikes with an increased interval between spikes, a phenomenon that could be associated with a greater influence of disynaptic retinal inhibition mediated by interneurons [44], [45]. Interestingly, this phenomenon has also been seen in the sustained response, both with pharmacological and cryogenical blockade of the cortex or in synchronized states of EEG [14]. The present study also shows that, after rTMS@1Hz in the cortex, the RF structure of X neurons seems to change, increasing the Gaussian amplitude in the periphery further than in the center.

### Functional implications of differential cortical effect

The present results reveal the ability of the cortex to differentially affect X and Y dLGN cells in a close relationship to the specific information carried by them. Y cells are mainly characterized by an initial transient response and a low sustained component. Thus, when a very brief visual stimulus is used (designed to emphasize the initial response) the cortex is able to influence Y cells response specifically by an effect on burst spikes suggesting an excitatory effect. When the cortical feedback is reduced, an increase of spikes in bursts and a decrease of tonic spikes is obtained without any change in the overall rate of firing, probably as a result of a sustained hyperpolarization.

On the other hand, rTMS@1Hz induces a delay and a decrease in peak response of X cells without any change in the proportion of burst and tonic spikes. This suggests a different corticofugal modulation. Thus, by suppressing excitatory inputs from the cortex (on relay cells, interneurons and perigeniculate nucleus) a decrease in Vm at a level insufficient to increase the burst response but, however, able to promote a hyperpolarization mediated by interneurons (and probably perigeniculate neurons) would facilitate the surround inhibitory effect. As a result, when retinal input is activated, an increase of interspike intervals is observed with a subsequent decrease and delay in the peak response. The RF analysis supports this hypothesis by showing a deviation of the excitation-inhibition balance toward the inhibitory component.

In summary, our data suggest that in physiological conditions the visual cortex controls in parallel two channels of information. In the faster route, corresponding to Y cells, it ensures an effective detection of new visual stimuli, selecting areas of interest of the image and producing a transient response with an optimal spike frequency to activate cortical cells. In addition, the cortex facilitates the firing of the X cells (slower) allowing an optimal time delay with respect to Y cells and, once new stimulus are detected, promote their synchronization for a detailed analysis of the image.

## Materials and Methods

### Experimental preparation

Six adult cats of either sex were prepared following standard procedures used in our laboratory for extracellular recordings in the visual pathway [17], [46]. Briefly, animals were anesthetized with isofluorane (5% for induction, 1.5–2% for surgery, and 0.5–1% for maintenance) in nitrous oxide (70%) and oxygen (30%). The trachea was cannulated, an intravenous line inserted, and a craniotomy performed for thalamic recordings. To prevent eye movements, animals were paralyzed with gallamine triethiodide (loading dose of 40 mg, maintenance 10 mg/kg/h i.v.) and held in a stereotaxic frame. End-tidal CO_2_ levels, electrocardiogram (EKG) waveform and intersystolic interval, and the electroencephalogram (EEG) were monitored continuously throughout the experiment. The rate and depth of artificial respiration was adjusted to maintain end-tidal CO_2_ at 3.8–4.2%; the level of isofluorane was chosen to achieve a state of light anesthesia. Once a stable state was reached, any variations in the monitored parameters (change in the amplitude/frequency of EEG waves or EKG frequency, fall or fluctuation in the intersystolic interval, and change in end-tidal CO_2_) commensurate with a change in the depth of anesthesia were compensated for by alterations in the level of isofluorane. Management of anesthesia was based upon spectrum of measures and follows the guidelines given by the UK Home Office. Wound margins were treated with lidocaine hydrochloride administered subcutaneously. The stereotaxic ear bars were coated with lidocaine gel. The eyes were treated with atropine methonitrate and phenylephrine hydrochloride, protected with zero-power contact lenses, and brought to focus on a semi-opaque tangent screen 57 cm distant using appropriate trial-case lenses. Visual stimuli were applied monocularly through 3 mm artificial pupils. To further reduce possible eye movement artifacts, rigid posts were fixed to the sclera and attached to the stereotaxic frame. At the end of experiments animals were killed by an anesthetic overdose. All animal work was conducted according to relevant national and international guidelines (Spanish Physiology Society and the International Council for Laboratory Animal Science and the European Union). The study was approved by the University of A Coruña Ethics Committee (CE-UDC30/1/09).

### Magnetic stimulation

The rTMS was carried out with a MagStim Rapid system (The MagStim Company Ltd, Whitland, UK) equipped with 2 boosters and applied on the occipital cortex of cats via a figure-of-eight coil (mean diameter: 25 mm). The midpoint of the coil was centered over the interhemispheric cranial suture at the level of area 17 (Horsley-Clarke antero-posterior: 0 to –6, Medio-lateral: 0) directly touching the exposed bone. The coil was fixed by a mechanical arm at an angle of about 60° (wings located laterally) with the handle pointing up and backwards. For this study, all rTMS parameters were optimized to produce cortical suppression in the region of cortex below the coil [10], [47]–[49]. The stimulation frequency utilized was one pulse per second, applied during a period of two minutes, hereinafter referred to as the rTMS@1Hz protocol. The intensity was set to 50% which, according to the data supplied by the manufacturer (http://www.magstim.co.uk), induces 1.5 T on the cortical surface (3 mm from the coil), and an electric field strength of 220 V/m, higher than those reported in other studies [50] but which effect, shown in the results, is consistent with those described by these authors.

The statistical significance of the changes induced by magnetic stimulation was determined by using paired and unpaired t-test or Wilcoxon test. The hypothesis of normality was checked using the Kolmogorov-Smirnov test. Results were deemed to be significant when P<0.05. Cluster analysis was carried out with the two-phase modality and by applying the Akaike criterion.

### Recording procedures

Tungsten microelectrodes (FHC) of 8–12 MÙ, mounted on a micromanipulator (FHC, Brunswick, ME) were introduced in the dLGN following stereotaxic coordinates (Horsley–Clark A6 L9). Neurons responding to visual stimulation of both eyes (binocular) with big RFs were classified as perigeniculate nucleus cells and were not considered. Neurons giving only monocular responses with a defined RF were considered for further recording and analysis. Cell type was identified, X or Y, on the basis of standard tests including the test of linearity of spatial summation, size and eccentricity of the receptive field, type of response to points of light, and presence of shift effect [51]–[54]. The visual response of the cell was recorded during four minutes: two minutes for control condition followed by two minutes for the simultaneous application of the rTMS@1Hz protocol. The entire protocol was run once per cell. The inter-recording interval was dependent upon the time devoted to search, isolate and characterize each cell, but usually was more than one hour.

### Visual stimulus

**Figure 8:**
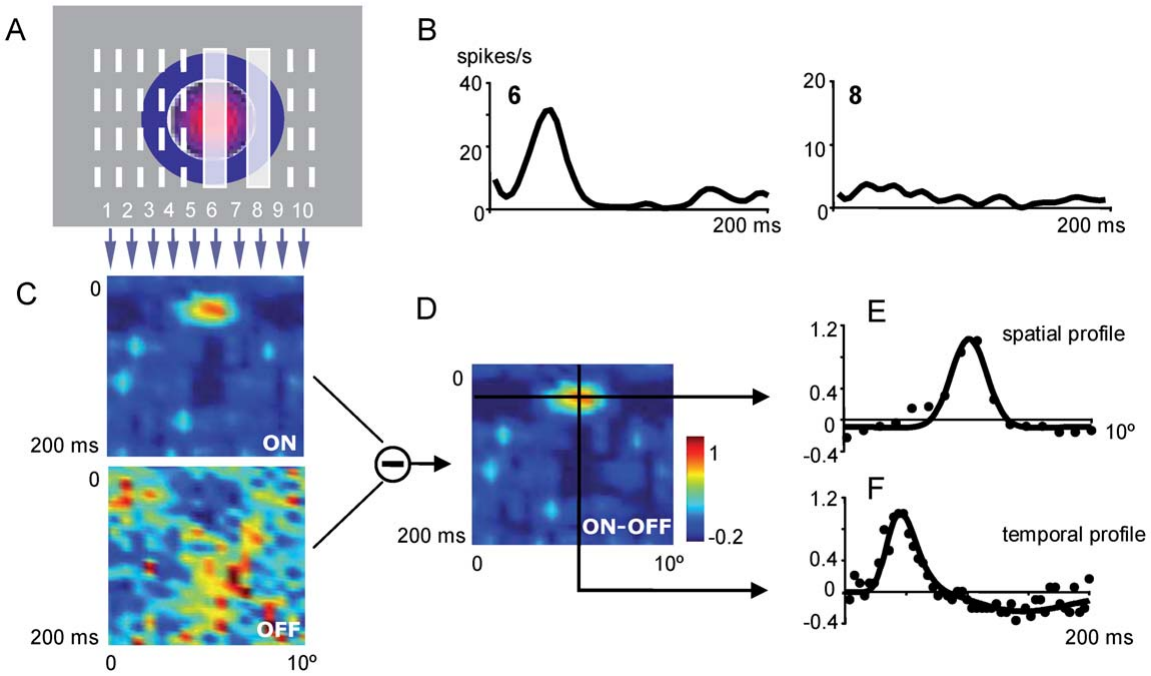
Spatiotemporal receptive field (STRF) profile obtained from a one-dimensional reverse correlation procedure. Visual stimulus is a randomized sequence of bright and dark bars centered at the dLGN cell's RF. ***A*** *,* Simplified figure (10 bars) showing two of the white bars (6 & 8) presented to the dLGN cell. ***B*** *,* Peristimulus time histogram (PSTH) from bars 6 & 8. Bin size: 2 ms. ***C*** *,* Spatiotemporal profiles obtained from white (ON) and dark (OFF) bar series. ***D*** *,* STRF obtained by subtracting OFF from ON spatiotemporal profile. ***E*** *,* Spatial RF profile obtained by slicing in the STRF through the RF center at peak response. ***F*** *,* Temporal profile derived by slicing in the STRF through the RF center. Mathematical approaches (solid curves) are obtained from real values (filled circles; see text for details).

The one-dimensional version of the reverse correlation algorithm [18], [55]–[58] (see below *Reverse correlation analysis*) was applied to evaluate the dLGN cells visual response. This protocol allows the quick generation of a RF map during the application of rTMS (the period was limited by the activation of the temperature-sensitive protection of the equipment). The transient response elicited in dLGN neurons was used to characterize the spatiotemporal dynamics of the RF as well as its response mode (see *Burst analysis*). In particular, the visual stimulus consisted in two groups of 20 white and 20 dark bars presented on a gray background and in a pseudorandom sequence. Each group of bars was arranged orderly and centered on the RF cell under study, as it is shown in Figure 8A. Stimuli were adapted to the preferred spatial frequency of the cells by changing the bar width. Bars had a length of 12° and a width between 0.4° and 1°, covering an area of +/− 6° of elevation and +/− 4° to 10° of azimuth respectively. Each bar was displayed for 40 ms with no direct correlation with the magnetic stimulation.

### Reverse correlation analysis

A diagram summarizing the procedure we used is shown on figure 8. Visual stimuli were displayed in an area centered over the dLGN cell's RF (Fig. 8A). Each spike was associated with the visual stimulus that preceded it for an interval of 200 ms, then a peristimulus time histogram (PSTH) for each bar was generated (Fig. 8B). PSTHs were ordered according to the position of the visual stimulus that elicited each one and normalized with respect to the maximum total value. As a result, spatial response profiles across the receptive field were obtained (Fig. 8C), both for ON stimulus (white bars) and OFF stimulus (black bars). A composite spatiotemporal receptive field (STRF) was then obtained subtracting the OFF from the ON response (Fig. 8D). Thus, for an ON cell, regions of the STRF excited by the bright and dark bars are shown as positive and negative values respectively. Figure 8E–F shows the spatial and temporal profiles obtained by slicing the STRF through the peak value as it is shown by arrows in Fig. 8D. Filled circles in Fig. 8E–F represent real values and solid curves show the mathematical fit for the spatial (difference of two Gaussian functions) [18], [59]–[61] and temporal (difference of two Gamma functions) [18], [62], [63] profiles. This process was applied to all STRFs to quantitatively characterize and compare different recordings. All curve fittings were performed with *cftool function (*MATLAB version R2006b, The *MathWorks*).

### Control experiments (visual cortical responses to local rTMS)

**Figure 9:**
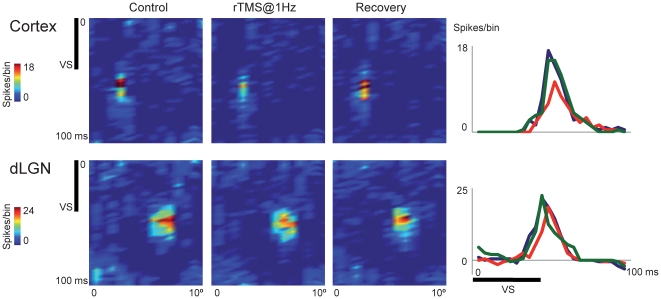
Example of the effect of rTMS on cortical cells. Top panel: rTMS stimulation produced a reduction in the peak response (red curve) compared to the pre rTMS values (blue curve). Green curve depicts the recovery of the response (bin = 4 ms). The cell was recorded in the deeper layers of V1. The effect of rTMS on a dLGN cell recorded simultaneously is also shown (bottom panel).

To gain insight into the effect of rTMS on V1, in previous control experiments we were able to record visual-driven activity simultaneously from cortical and dLGN cells during rTMS stimulation at 1 Hz. Electrodes to record cortical spikes were either advanced during the experiment at an oblique angle (avoiding the stimulating coil) or cemented in place (tip located approximately in layer VI) prior to positioning of the TMS coil. Visual stimulus was the same one used to study dLGN cells; the orientation of the bars was the optimal for each cortical cell. We did not pretend to systematically map the spatial extent of the cortical suppression zone; however, it is likely, given the size of the cat brain and the spatial resolution of rTMS that our stimulation effectively covered the primary visual cortex and that the effect was uniform across this region. The histogram in figure 9 shows the visual response of both, a dLGN neuron and a deep layer cortical cell (presumably a layer 6 neuron), before (blue line), during rTMS (red line) and after rTMS (green line).

### Intervalogram analysis

**Figure 10:**
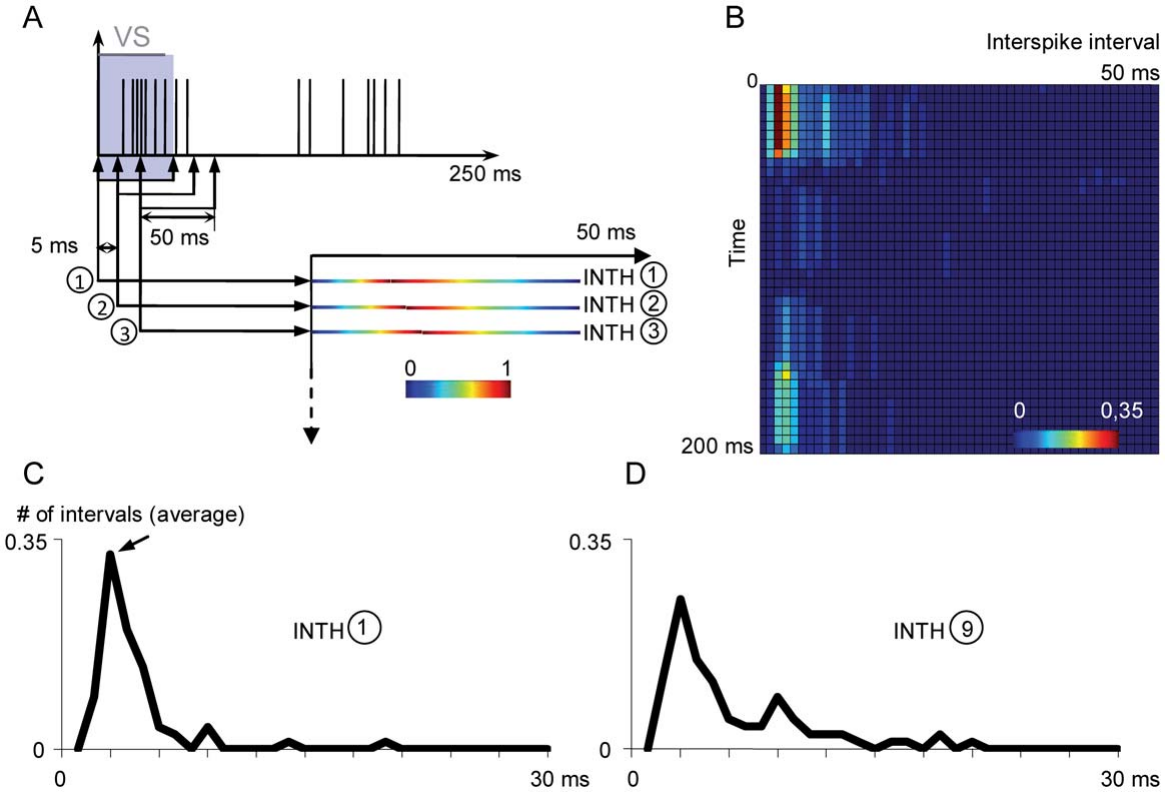
Construction of intervalograms. ***A***, Spikes elicited by a dark bar (VS) presented on RF center of a Y off dLGN cell. Interval distributions are computed for small time windows of 50 ms shifted 5 ms each other along the time axis. An inter-spike interval histogram (INTH) is calculated for each window (1, 2, 3, … 40) by adding all interval distributions obtained from each dark bar presented on RF center of the dLGN cell. INTHs are plotted as a colour-scaled horizontal pixel line and all the intervalogram is normalized to its maximum value. ***B***, Average intervalogram for all Y dLGN cells. The temporal resolution for the horizontal axis is 1 ms and the maximum value is limited by the time window, i.e. 50 ms. The temporal resolution for the vertical axis is determined by the interval between time windows, i.e. 5 ms and maximum value is 200 ms. ***C*** *,* First INTH of the average intervalogram showed in ***B***. ***D*** *,* INTH from the average intervalogram at the end of the VS.

The intervalogram method was introduced by Funke and Wörgötter [44] to study the sustained phase of cat dLGN cell responses. Basically, an intervalogram shows the variation of the distribution of interspike intervals in the cell response. Figure 10A shows schematically the response of a cell to one bar displayed on the RF center. Firstly, in a time window of 50 ms (see below) starting at t = 0, a first interspike interval histogram (INTH) was obtained for the spikes within the window. The second INTH was obtained for a time window displaced 5 ms after the first window (5 ms corresponds to the 10% of the total duration of the window according to the criterion of Funke and Wörgötter) [44]. Remaining INTH's were obtained moving the window every 5 ms to complete the total period of analysis (250 ms). Arranging INTH's in a two dimensional array allowed us to detect changes on interval distribution over time whenever the cell was stimulated. Due to the visual stimulus properties, intervalograms illustrate changes during the transient phase of the response. Since each visual stimulus generated an intervalogram, an average intervalogram for a given number of stimuli was obtained. Figure 10B shows an average intervalogram for a Y OFF cell obtained after the presentation of 72 black bars on the RF center. Figure 10C shows the first mean INTH of that intervalogram, obtained immediately after the onset of the visual stimulus. The cell responded preferentially with spikes separated 3 ms (arrow). Figure 10D shows the mean 9^th^ INTH, calculated from a time window starting at the end of the visual stimulus (40 ms); even though the preferred frequency response is maintained, the 9^th^ INTH is more distributed than the first INTH i.e., spikes with longer intervals begin to appear.

In general, when intervalograms are used, some details must be taken into account. There is a compromise between the time window size and the characteristics of the stimulus used. A large time window has the advantage of sampling a higher number of spikes and thus generating INTHs with a higher resolution, but it is not optimal to analyze transient responses. In contrast, smaller time windows generate intervalograms with a greater temporal resolution, but with fewer spikes used to compute each INTH. We chose 50 ms because it was optimal to analyze the transient phase of dLGN cell responses. Regarding the analysis of intervalograms, due to the fact that each point in the intervalogram represents how many times a value of interspike interval is repeated in certain time window, the peak value of intervalograms before and after applying rTMS can move in a 2D plane i.e., a cell can either change its preferred firing frequency, or the instant it reaches the peak, or both parameters at once. The latter is a problem when applying statistics and it is the reason to read intervalograms in a qualitative way.

### Burst analysis

Burst analysis was carried out to unveil changes in the response mode of dLGN neurons. Trains of action potentials preceded by a silence period of at least 50 ms and whose interspike intervals were less than 4 ms were considered as bursts [23]. All other spikes were considered as tonic response.

## References

[1] Kalil RE, Chase R (1970). Corticofugal influence on activity of lateral geniculate neurons in the cat.. J Neurophysiol.

[2] Singer W (1977). Control of thalamic transmission by corticofugal and ascending reticular pathways in the visual system.. Physiol Rev.

[3] Geisert EE, Langsetmo A, Spear PD (1981). Influence of the cortico-geniculate pathway on response properties of cat lateral geniculate neurons.. Brain Res.

[4] McClurkin JW, Marrocco RT (1984). Visual cortical input alters spatial tuning in monkey lateral geniculate nucleus cells.. J Physiol (Lond).

[5] Murphy PC, Sillito AM (1987). Corticofugal feedback influences the generation of length tuning in the visual pathway.. Nature.

[6] Sillito AM, Cudeiro J, Jones HE (2006). Always returning: Feedback and sensory processing in visual cortex and thalamus.. Trends Neurosci.

[7] Hallett M (2000). Transcranial magnetic stimulation and the human brain.. Nature.

[8] Sack AT (2006). Transcranial magnetic stimulation, causal structure–function mapping and networks of functional relevance.. Curr Opin Neurobiol.

[9] Mix A, Benali A, Eysel UT, Funke K (2010). Continuous and intermittent transcranial magnetic theta burst stimulation modify tactile learning performance and cortical protein expression in the rat differently.. Eur J Neurosci.

[10] de Labra C, Rivadulla C, Grieve K, Marino J, Espinosa N (2007). Changes in visual responses in the feline dLGN: Selective thalamic suppression induced by transcranial magnetic stimulation of V1.. Cereb Cortex.

[11] Pasley BN, Allen EA, Freeman RD (2009). State-dependent variability of neuronal responses to transcranial magnetic stimulation of the visual cortex.. Neuron.

[12] Wang W, Jones HE, Andolina IM, Salt TE, Sillito AM (2006). Functional alignment of feedback effects from visual cortex to thalamus.. Nat Neurosci.

[13] Funke K, Nelle E, Li B, Wörgötter F (1996). Corticofugal feedback improves the timing of retino-geniculate signal transmission.. Neuroreport.

[14] Wörgötter F, Suder K, Zhao Y, Kerscher N, Eysel UT (1998). State-dependent receptive-field restructuring in the visual cortex.. Nature.

[15] Godwin DW, Vaughan JW, Sherman SM (1996). Metabotropic glutamate receptors switch visual response mode of lateral geniculate nucleus cells from burst to tonic.. J Neurophysiol.

[16] Fanselow EE, Sameshima K, Baccala LA, Nicolelis M (2001). Thalamic bursting in rats during different awake behavioral states.. Proc Natl Acad Sci USA.

[17] Rivadulla C, Martinez L, Grieve KL, Cudeiro J (2003). Receptive field structure of burst and tonic firing in feline lateral geniculate nucleus.. J Physiol (Lond).

[18] Cai D, Deangelis GC, Freeman RD (1997). Spatiotemporal receptive field organization in the lateral geniculate nucleus of cats and kittens.. J Neurophysiol.

[19] Chen R, Classen J, Gerloff C, Celnik P, Wassermann EM (1997). Depression of motor cortex excitability by low-frequency transcranial magnetic stimulation.. Neurology.

[20] Alitto HJ, Weyand TG, Usrey WM (2005). Distinct properties of stimulus-evoked bursts in the lateral geniculate nucleus.. J Neurosci.

[21] Lesica NA, Weng C, Jin J, Yeh C, Alonso J (2006). Dynamic encoding of natural luminance sequences by LGN bursts.. PLoS Biol.

[22] Wang X, Wei Y, Vaingankar V, Wang Q, Koepsell K (2007). Feedforward excitation and inhibition evoke dual modes of firing in the cat's visual thalamus during naturalistic viewing.. Neuron.

[23] Lu SM, Guido W, Sherman SM (1992). Effects of membrane voltage on receptive field properties of lateral geniculate neurons in the cat: Contributions of the low-threshold Ca2+ conductance.. J Neurophysiol.

[24] Reinagel P, Godwin D, Sherman SM, Koch C (1999). Encoding of visual information by LGN bursts.. J Neurophysiol.

[25] Smith GD, Sherman SM (2002). Detectability of excitatory versus inhibitory drive in an integrate-and-fire-or-burst thalamocortical relay neuron model.. J Neurosci.

[26] Grubb MS, Thompson ID (2005). Visual response properties of burst and tonic firing in the mouse dorsal lateral geniculate nucleus.. J Neurophysiol.

[27] Grieve KL, Rivadulla C, Cudeiro J (2009). Mixed burst and tonic firing in the thalamus: A study in the feline lateral geniculate nucleus in vivo.. Brain Res.

[28] Weyand TG, Boudreaux M, Guido W (2001). Burst and tonic response modes in thalamic neurons during sleep and wakefulness.. J Neurophysiol.

[29] Waleszczyk WJ, Bekisz M, Wróbel A (2005). Cortical modulation of neuronal activity in the cat's lateral geniculate and perigeniculate nuclei.. Exp Neurol.

[30] Zhan XJ, Cox CL, Rinzel J, Sherman SM (1999). Current clamp and modeling studies of low-threshold calcium spikes in cells of the cat's lateral geniculate nucleus.. J Neurophysiol.

[31] Mukherjee P, Kaplan E (1995). Dynamics of neurons in the cat lateral geniculate nucleus: in vivo electrophysiology and computational modeling.. J Neurophysiol.

[32] Fukuda Y, Stone J (1976). Evidence of differential inhibitory influences on X- and Y-type relay cells in the cat's lateral geniculate nucleus.. Brain Res.

[33] Berardi N, Morrone MC (1984). Development of gamma-aminobutyric acid mediated inhibition of X cells of the cat lateral geniculate nucleus.. J Physiol (Lond).

[34] Montero VM, Zempel J (1985). Evidence for two types of GABA-containing interneurons in the A-laminae of the cat lateral geniculate nucleus: A double-label HRP and GABA-immunocytochemical study.. Exp Brain Res.

[35] Lindström S, Wróbel A (1990). Private inhibitory systems for the X and Y pathways in the dorsal lateral geniculate nucleus of the cat.. J Physiol (Lond).

[36] Livingstone M, Hubel D (1987). Psychophysical evidence for separate channels for the perception of form, color, movement, and depth.. J Neurosci.

[37] Nassi JJ, Callaway EM (2009). Parallel processing strategies of the primate visual system.. Nat Rev Neurosci.

[38] Briggs F, Usrey WM (2009). Parallel processing in the corticogeniculate pathway of the macaque monkey.. Neuron.

[39] Vidnyanszky Z, Hamori J (1994). Quantitative electron microscopic analysis of synaptic input from cortical areas 17 and 18 to the dorsal lateral geniculate nucleus in cats.. J Comp Neurol.

[40] Murphy P, Sillito A (1996). Functional morphology of the feedback pathway from area 17 of the cat visual cortex to the lateral geniculate nucleus.. J Neurosci.

[41] Murphy PC, Duckett SG, Sillito AM (2000). Comparison of the laminar distribution of input from areas 17 and 18 of the visual cortex to the lateral geniculate nucleus of the cat.. J Neurosci.

[42] Sillito AM, Kemp JA (1983). The influence of GABAergic inhibitory processes on the receptive field structure of X and Y cells in cat dorsal lateral geniculate nucleus (dLGN).. Brain Res.

[43] Cleland BG, Lee BB (1985). A comparison of visual responses of cat lateral geniculate nucleus neurones with those of ganglion cells afferent to them.. J Physiol (Lond).

[44] Funke K, Wörgötter F (1995). Temporal structure in the light response of relay cells in the dorsal lateral geniculate nucleus of the cat.. J Physiol (Lond).

[45] Funke K, Wörgötter F (1997). On the significance of temporally structured activity in the dorsal lateral geniculate nucleus (LGN).. Prog Neurobiol.

[46] Rivadulla C, Martinez LM, Varela C, Cudeiro J (2002). Completing the corticofugal loop: A visual role for the corticogeniculate type 1 metabotropic glutamate receptor.. J Neurosci.

[47] Wassermann EM, Cohen LG, Flitman SS, Chen R, Hallett M (1996). Seizures in healthy people with repeated "safe" trains of transcranial magnetic stimuli.. Lancet.

[48] Maeda F, Keenan JP, Tormos JM, Topka H, Pascual-Leone A (2000). Modulation of corticospinal excitability by repetitive transcranial magnetic stimulation.. Clin Neurophysiol.

[49] Aydin-Abidin S, Moliadze V, Eysel UT, Funke K (2006). Effects of repetitive TMS on visually evoked potentials and EEG in the anaesthetized cat: Dependence on stimulus frequency and train duration.. J Physiol (Lond).

[50] Moliadze V, Zhao Y, Eysel U, Funke K (2003). Effect of transcranial magnetic stimulation on single-unit activity in the cat primary visual cortex.. J Physiol (Lond).

[51] Enroth-Cugell C, Robson JG (1966). The contrast sensitivity of retinal ganglion cells of the cat.. J Physiol (Lond).

[52] Cleland BG, Dubin MW, Levick WR (1971). Sustained and transient neurones in the cat's retina and lateral geniculate nucleus.. J Physiol (Lond).

[53] Shapley R, Hochstein S (1975). Visual spatial summation in two classes of geniculate cells.. Nature.

[54] Derrington AM, Fuchs AF (1979). Spatial and temporal properties of X and Y cells in the cat lateral geniculate nucleus.. J Physiol (Lond).

[55] Jones JP, Palmer LA (1987). The two-dimensional spatial structure of simple receptive fields in cat striate cortex.. J Neurophysiol.

[56] Palmer LA, Jones JP, Stepnoske RA, Leventhal AG (1991). Striate receptive fields as linear filters: Characterization in two dimensions of space.. Vision and visual dysfunction.

[57] DeAngelis GC, Ohzawa I, Freeman RD (1993). Spatiotemporal organization of simple-cell receptive fields in the cat's striate cortex. I. general characteristics and postnatal development.. J Neurophysiol.

[58] DeAngelis GC, Ohzawa I, Freeman RD (1993). Spatiotemporal organization of simple-cell receptive fields in the cat's striate cortex. II. linearity of temporal and spatial summation.. J Neurophysiol.

[59] Rodieck RW (1965). Quantitative analysis of cat retinal ganglion cell response to visual stimuli.. Vision Res.

[60] Enroth-Cugell C, Robson JG, Schweitzer-Tong DE, Watson AB (1983). Spatio-temporal interactions in cat retinal ganglion cells showing linear spatial summation.. J Physiol (Lond).

[61] Dawis S, Shapley R, Kaplan E, Tranchina D (1984). The receptive field organization of X-cells in the cat: Spatiotemporal coupling and asymmetry.. Vision Res.

[62] Adelson EH, Bergen JR (1985). Spatiotemporal energy models for the perception of motion.. J Opt Soc Am A.

[63] Watson AB, Ahumada AJ (1985). Model of human visual-motion sensing.. J Opt Soc Am A.

